# Impaired feedforward control of movements in pianists with focal dystonia

**DOI:** 10.3389/fneur.2022.983448

**Published:** 2022-08-12

**Authors:** Ken Takiyama, Shuta Mugikura, Shinichi Furuya

**Affiliations:** ^1^Department of Electrical Engineering and Computer Science, Tokyo University of Agriculture and Technology, Koganei, Japan; ^2^Sony Computer Science Laboratories Inc. (Sony CSL), Tokyo, Japan; ^3^Sophia University, Tokyo, Japan; ^4^Institute for Music Physiology and Musicians' Medicine, Hannover University of Music, Drama, and Media, Hannover, Germany; ^5^NeuroPiano Institute, Kyoto, Japan

**Keywords:** focal hand dystonia (FHD), maladaptation, feedforward control, skill learning, data-driven method, task-relevant dimension

## Abstract

Learning accurate and fast movements typically accompanies the modulation of feedforward control. Nevertheless, it remains unclear how motor skill learning modulates feedforward control, such as through maladaptation of the sensorimotor system by extensive training (e.g., task-specific dystonia). Here, we examined the modulation of feedforward control through motor skill learning while focusing on the motion of piano playing at either a natural tempo or the fastest tempo. The current study compared the kinematics and keypress data among individuals in three groups: healthy and well-trained pianists (i.e., subjects with skill learning), non-musicians (i.e., subjects without skill learning), and patients with focal-hand dystonia (FHD) (i.e., subjects with maladaptation by skill learning). Compared to healthy pianists, patients with FHD showed impairment in some feedforward motion components that are relevant to classifying the two playing tempi. However, while focusing on motion components that are irrelevant to the tempo classification, patients with FHD showed movements comparable to those of healthy pianists. Furthermore, patients with FHD demonstrated significantly slower movement times than healthy pianists. Our results suggest that maladaptation by skill learning affects parts of feedforward control rather than its entirety. Nevertheless, the affected feedforward components are relevant to performing movements as fast as possible, which may underlie the speed dependence of dystonic symptoms.

## Introduction

Sophisticated and skilled performances in music and sports have long attracted and excited people. Professional soccer players demonstrate either better accuracy or speed in passing, shooting, and dribbling than amateurs (1). Top-level musicians play musical instruments at a surprisingly fast speed with great accuracy (2, 3). Expert programmers can judge the correctness of program codes faster and more accurately than novices (novice/expert differences in programming skills). As illustrated by these examples, accuracy and speed are key components of skilled motion, indicating that motor skill is defined as the ability to perform accurate and fast movements (4, 5).

We acquire motor skills through practice, a learning process that is referred to as skill learning. Previous studies have examined the properties of skill learning through laboratory settings. In a task to perform arm-reaching movements within restricted curved areas, skill learning induced faster movement times and higher success rates by reducing trial-to-trial variability of the hand trajectory (6). In a virtual throwing task, not only did the variability decrease but also the mean kinematic parameters approached the optimal parameters (7). In finger-tapping tasks, training facilitated improvements in accuracy and speed, and associated neural plasticity has also been reported (8, 9). Skill learning thus facilitates the improvement of (at least) accuracy and speed in motor control.

Although it is widely known that skill learning improves speed and accuracy in motor control, the details about such learning remain under investigation; specifically, how skill learning modifies feedforward and feedback control remains unclear (4). Motor control has been classified into at least two categories: feedforward and feedback control. Feedforward control relies heavily on predictive information about visual, proprioceptive, or external environmental domains (10) but not on sensory information. These features of feedforward control enable us to generate fast movements by overcoming fatal sensory delays. Feedforward control is evident in actions such as playing musical instruments with the fastest tempo (2, 3), swinging a baseball bat, or rapid arm-reaching movements under environmental changes (11–14). In contrast to feedforward control, feedback control depends on sensory information with a time delay, such as a motor response of 160 msec after detecting changes in visual information (15). Feedback control plays important roles in correcting online movement errors. In sum, especially at the beginning of movements, feedforward control is more prominent than feedback control because sensory information is not yet available for movement correction. Subsequently, feedforward and feedback control play essential roles. A previous study reported that skill learning affects feedback control in arm-reaching movements after 3 days of training (6). A contrasting viewpoint is that skill learning facilitates reliance on feedforward control, especially in young people (16, 17). It thus remains unclear how skill learning affects feedforward and feedback control.

Here, we clarified the influence of skill learning on feedforward and feedback control while focusing on the motions to play the piano at the natural or fastest tempo. In particular, we focused on how skill learning affects feedforward control because the difference in motions between the natural and fastest tempi appears around the beginning of motions (18) [cf. (19)]. Additionally, we compared the motions performed to play the piano between professionals and non-musicians to address the influence of skill learning. Because a larger amount of training enables us to achieve highly skilled motions (20), professionals who practice longer than amateurs can be an ideal model to address this issue. In contrast to simple movements, such as arm-reaching movements, the comparison between the professional pianists and non-musicians allows us to investigate clearly the influence that skill learning exerts on motor control not just over a few days but across several years. Playing the piano at the fastest tempo requires skill to achieve fast and accurate movements. Because piano playing at a submaximal tempo does not require particular skills to achieve fast movements, it can work as a baseline of motor skill in each participant. Based on a previous finding (18), the difference between the natural and fastest tempi should appear primarily around the initiation of playing. We thus evaluated the motion components relevant to the classification of tempo into its fastest and natural components while expecting to quantify the effects of skill learning on feedforward control. To extract the non-trivial classification-relevant motion components, we utilized a data-driven technique (21–23).

The current study also addressed another aspect of skill learning: maladaptation. Skill learning has pros and cons. A positive aspect is its facilitation of motions with accuracy and speed of movements, whereas a negative aspect is maladaptation through overtraining. Maladaptation causes painless loss of skill in repetitively trained tasks. For musicians, focal hand dystonia (FHD) is one representative form of maladaptive skill learning (24). FHD entails involuntary muscle convulsions while performing trained movements (25). Although the causes of FHD are still being investigated, a possible triggering factor is the repetitive training of precise motions (5). Individuals with FHD demonstrate various symptoms, such as loss of fine motor control (26), atypical joint coordination (27), and impaired non-motor functions (28).

It is still unclear what pathological mechanisms underlie FHD symptoms. Specifically, one unsolved question is how the maladaptive effects of skill learning affect feedforward and feedback control. Some previous findings have identified the impairment of sensory-motor integration in patients with FHD (29–31). For example, the patients showed overreacting grip force when lifting an object (32), indicating an incorrect estimation of sensory information. These findings supported the impairment of feedback control in patients with FHD. The same study mentioned an intact predictive response in grip force while lifting objects (32). Because predictive motor responses primarily reflect feedforward control, we can expect intact feedforward control in patients with FHD. In contrast, patients with FHD show deficits in motor imagery (33, 34) that possibly indicate a deficit of predictive function and feedforward control. Overall, although feedback control is likely to be impaired in patients with FHD, the effects of FHD on feedforward control are still unclear. The current study focuses on how maladaptation affects feedforward components by comparing skillful finger movements at the fastest tempo to the same movements at a natural tempo.

We aimed to clarify the influence of skill learning, especially on feedforward control, based on the comparison among non-musicians (i.e., subjects without skill learning in piano playing), professionals (i.e., subjects with long-lasting skill learning in piano playing), and patients with FHD (i.e., subjects with maladaptive influence *via* skill learning in piano playing). The comparison of the motions performed to play the piano at the fastest and natural tempi permits us to probe the influence of skill level on feedforward control. By focusing especially on the motion features relevant to classifying tempo into the natural and fastest categories, particularly when beginning to play (18), we discuss the influence of skill learning on feedforward control. The current study focuses on behavioral aspects because these are essential not only for providing insights into neural mechanisms of skill learning and maladaptation but also for clinical application, such as for the rehabilitation and diagnosis of FHD.

First, the current study focuses on the difference in joint angular kinematics among non-musicians, professionals, and FHD patients while playing one of the simplest piano pieces. Data-driven techniques allowed us to assess non-trivial motion components that are relevant to classifying tempo into natural and fastest components (21–23). Although intact predictive control in patients with FHD (32) indicated few differences in feedforward control among the groups, impaired functions in motor imagery (33) suggested the opposite results. To verify these predictions, we examined the difference in kinematics among participants in the three groups.

Second, we compared speed and accuracy among non-musicians, professionals, and patients with FHD based on the keypress data derived from the musical instrumental digital interface (MIDI). For a fair comparison of joint angle data between tempi, we normalized movement time to be the same across the two tempi. The normalized joint angle data thus do not provide information about movement time. In addition, joint angle data do not directly reflect whether each keypress is correct. We thus analyzed keypress data to discuss how skill learning affects speed and accuracy in playing the piano. Keypress data include the timing of the individual keypresses, which yields the interstroke interval (i.e., movement time). The data incorporate an overview of motion data rather than detailed kinematic information. A feasible result is impairment of temporal accuracy in patients with FHD. Additionally, we can expect groupwise differences in the interstroke interval. In patients with a different form of dystonia (i.e., idiopathic torsion dystonia), movement time tends to be slower than that for intact individuals (35). Although we should care about whether different types of dystonia belong to the same class of disease (36), a plausible result is slower movement time in patients with FHD compared to that of individuals in the other groups. To confirm these speculations, the current study compared keypress data among the three groups, which to the best of our knowledge, has never been investigated. We also expected the difference in the keypress data to be a clinical marker of FHD.

Finally, the current study compared healthy pianists and patients with FHD when playing slightly difficult pieces of music to examine the influence of the difficulty of the pieces. Because the pieces were too difficult for non-musicians to play at the fastest tempo, the current study compared pianists with and without FHD.

## Results

**Figure 1:**
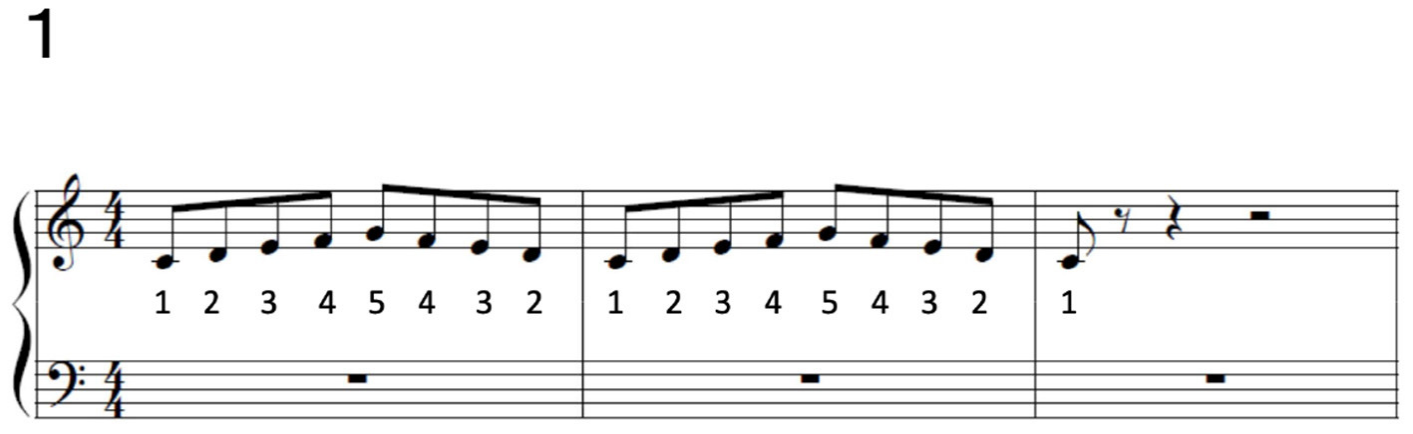
Piece #1. The numbers below musical notes indicate the fingers to use to play each note: 1 indicates the thumb, 2 indicates the index finger, 3 indicates the middle finger, 4 indicates the ring finger, and 5 indicates the little finger.

We analyzed the joint angle data of 13 healthy pianists, 23 patients with FHD, and 28 non-musicians (see STAR Methods for details). All the subjects played a short, simple piece of music [Figure 1 (piece #1)] at both the natural and fastest tempi. Both healthy pianists and patients with FHD played eight extra-short pieces of music at both a natural tempo and the fastest tempo, as shown in the Supplementary material (pieces #2–#9). For the simplest piece of music, all subjects were required to strike the five adjacent keys with the thumb, index finger, middle finger, ring finger, and little finger in order or in reverse order. Because non-musicians succeeded in playing only the simplest piece of music, we compared the three groups based on that piece. The extra pieces of music for healthy pianists and patients with FHD included various types of finger movements, such as the “thumb-under,” in which the thumb crosses index and middle fingers under the palm while depressing the piano with the middle finger.

We measured 15 joint angles, as listed in Table 1. All the joint angles were standardized such that the mean and standard deviation of each joint across time were 0 and 1, respectively. The number of time frames was also normalized to 200 across all the conditions to apply the logistic regression mentioned below. The normalizations enabled us to fairly compare all the measured kinematic parameters in playing musical pieces at the fastest tempo to the data acquired when musical pieces were played at a natural tempo. Of note, the standardizations did not affect the original motion data because linear reformatting enabled us to recover the original data (22).

**Table 1:** Measured joint angles.

T-TMJ	T-MP	T-IP	T-Abd	I-MP
I-PIP	M-MP	M-PIP	IM-Abd	R-MP
R-PIP	MR-Abd	L-MP	L-PIP	RL-Abd

T-, I-, M-, R-, L-, indicate the joint angles of the thumb, index finger, middle finger, ring finger, and little finger, respectively. IM-, MR-, and RL- indicate the abduction/adduction between the two adjacent fingers. TMJ indicates the trapeziometacarpal joint, MP indicates metacarpophalangeal, IP indicates interphalangeal, Abd indicates abduction, and PIP indicates proximal-phalangeal.

The current study analyzed joint angle data at a natural tempo $x_{n,i}\in {\mathbb{R}}^{1\times 3000}$ and the fastest tempo $x_{m,j}\in {\mathbb{R}}^{1\times 3000}$ (*i* = 1, …, *T*_*n*_, *j* = 1, …, *T*_*m*_, where *T*_*n*_ indicated the number of trials at a natural tempo, and *T*_*m*_ meant the number of trials at the fastest tempo). In addition, there were 3,000 rows of *x*_*n,i*_ and *x*_*m,j*_, which denoted 15 (the number of joint angles) multiplied by 200 (the number of normalized time frames). In other words, joint angle data were vectorized in each trial. For convenience, let us define $X=\left(\begin{array}{c} X_{n}\\ X_{m} \end{array}\right)\in {\mathbb{R}}^{\left(T_{n}+T_{m}\right)\times \;3000}$, where $X_{n}={\left(x_{n,1}^{T},...,x_{n,T_{n}}^{T}\right)}^{T}\in {\mathbb{R}}^{T_{n}\times 3000}$, $X_{m}={\left(x_{m,1}^{T},...,x_{m,T_{m}}^{T}\right)}^{T}\in {\mathbb{R}}^{T_{m}\times 3000}$, and (·)^*T*^ denotes the transpose of the vector.

Additionally, we defined target data $d={\left(0,...,0,1,...,1\right)}^{T}\in {\mathbb{R}}^{\left(T_{n}+T_{m}\right)\times 1}$ including *T*_*n*_ 0 s and *T*_*m*_ 1 s. *d*_*k*_ = 0 and *d*_*k*_ = 1 indicate that the *k*th trial occurred at a natural tempo or the fastest tempo, respectively (*k* = 1, ..., *T*_*n*_+*T*_*m*_).

To extract the kinematic features to play pieces of music at the fastest tempo, we utilized logistic regression with ridge regularization (37). The primary purpose of logistic regression is to find a sigmoidal function *f*(*x*_*k*_ ) ∈ [0, 1], where $x_{k}\in {\mathbb{R}}^{1\times 3000}$ indicates the motion data at the *k*th trial or *k*th row of X (*k* = 1, ..., *T*_*n*_+*T*_*m*_). The sigmoidal function *f*(*x*_*k*_) represents the probability that *x*_*k*_ can be classified as the fastest tempo, i.e., we estimated *f*(*x*_*k*_) to satisfy *f*(*x*_*k*_) = *p*(*d*_*k*_ = 1) and 1−*f*(*x*_*k*_) = *p*(*d*_*k*_ = 0). Throughout the current study, $f\left(x_{k}\right)=\frac{1}{1+\exp\left(-w_{0}-x_{k}w\right)}$, where *w*_0_ is an intercept, and *w* ∈ ℝ^3000 × 1^ indicates weight coefficients to calculate the sigmoid function *f*(*x*_*k*_). The current study utilized a hard decision boundary with *f*(*x*_*k*_) = 0.5, indicating that *x*_*k*_ was estimated to be associated with the fastest tempo and a natural tempo, when *f*(*x*_*k*_) ≥ 0.5 or not, respectively. The weight coefficients *w* signify to what extent each joint angle at each time frame contributed to the classification of the tempo as a natural tempo or the fastest one. We estimated *w* and *w*_0_ to minimize the prediction error between the predicted and actual target data.

**Figure 2:**
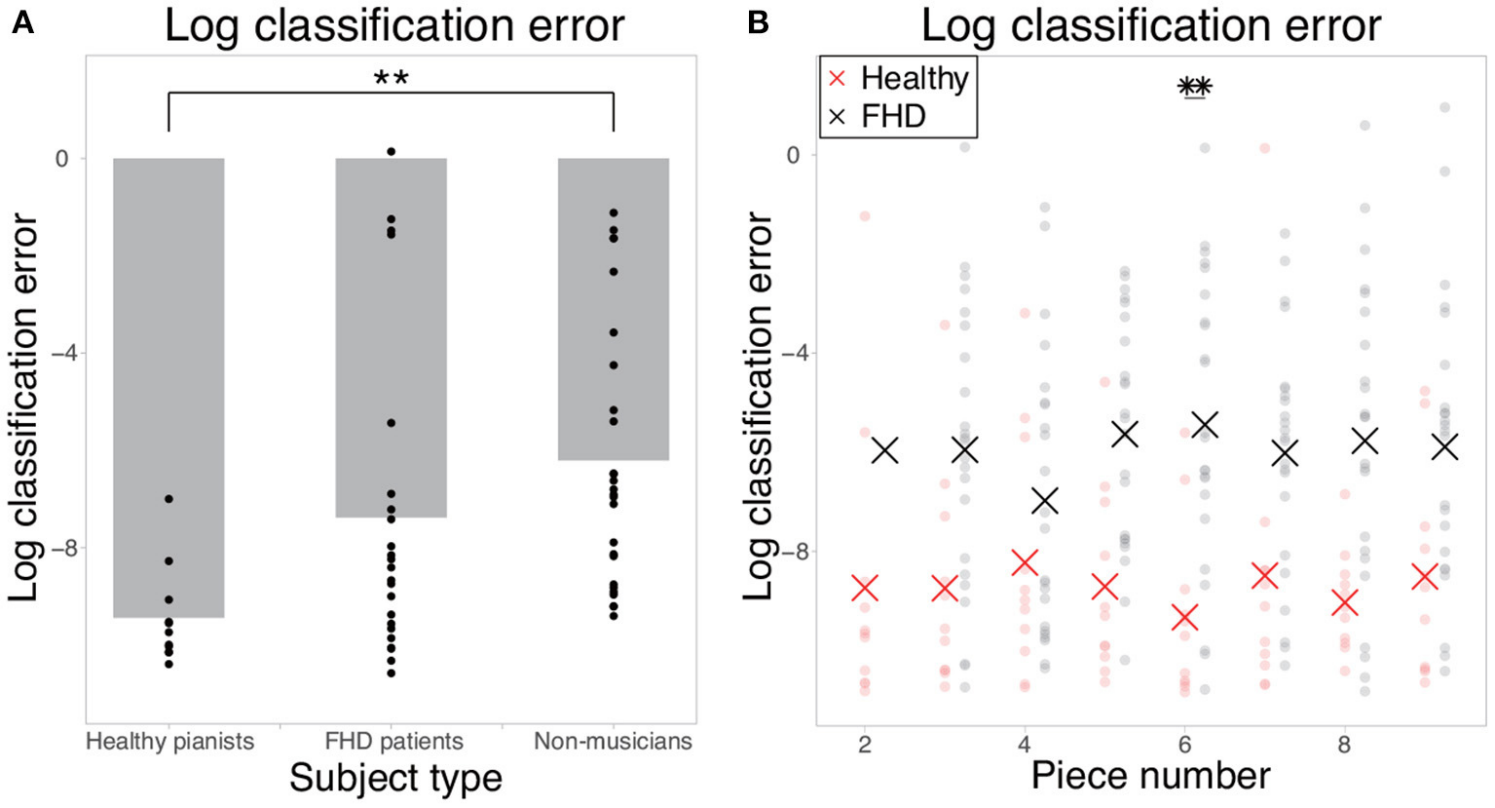
Logarithmic classification errors in classifying finger joint data as that collected when individuals were playing at the fastest tempo or a natural tempo. **(A)** The logarithmic classification error in piece #1. Each dot indicates the error in each subject, and the gray bar indicates the mean classification error in each group. Double asterisks indicate significant differences with *p* < 0.01. **(B)** The logarithmic classification error in the cases of pieces #2-#9. Each red and black dot indicates the error in each healthy pianist and patient with FHD, respectively. Red and black asterisks indicate the mean logarithmic classification error in each piece. Double asterisks indicate significant differences with *p* < 0.01.

If motion data included information that would allow perfect separation of the two tempi, the classification error would be 0, or the logarithmic classification error would be close to −∞. If motion data did not include any relevant information for classification, the classification error would be 1, or the logarithmic classification error would be close to 0. The classification error in piece #1 (i.e., the simplest piece) was 1.53 × 10^−4^± 2.60 × 10^−4^, 8.20 × 10^−3^ ± 0.248, and 4.25 × 10^−3^ ± 8.91 × 10^−2^ in healthy pianists, patients with FHD, and non-musicians, respectively (Figure 2, Figure 2 shows logarithmic classification error). There was a significant difference in the logarithmic classification error between the healthy pianists and non-musicians [p = 0.0052 (corrected)]. In pieces #2-#9 (i.e., pieces too difficult for non-musicians to play at the fastest tempo), the averaged classification errors in each piece were 1.82 × 10^−4^- 0.105 in healthy pianists and 0.0616-0.128 in patients with FHD. There was a significant main group effect of the logarithmic classification error [F (1, 32) = 18.4, p = 1.55 × 10^−4^], no significant main effect of piece number [F (7, 224) = 0.159, *p* = 0.993], and no significant interaction between group and piece number [F (7, 224) = 0.760, *p* = 0.621] (see the STAR Methods section for statistical analysis details). In the comparison between healthy pianists and patients with FHD in each piece, there was a significant difference in the logarithmic classification error in piece #6 (*p* = 0.0280) and no significant difference in other pieces (*p* > 0.0617). The logistic regression enabled us to extract the motion components relevant to classifying the two tempi compared to a random classifier with a classification error of 0.5 or a logarithmic classification error of −0.301.

**Figure 3:**
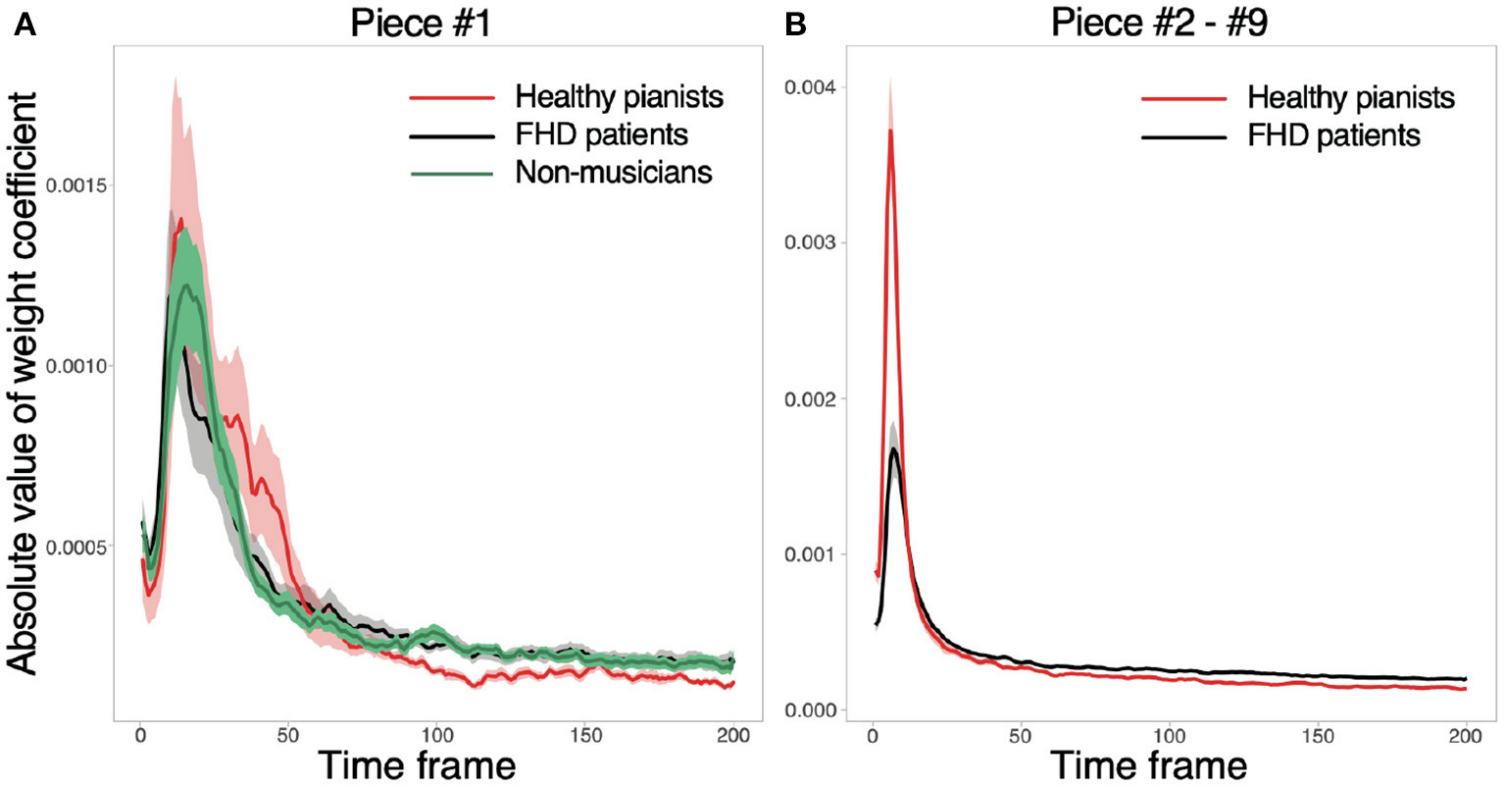
Absolute values of *w* in each time frame. **(A)** The absolute values for piece #1. Red, black, and gray solid lines indicate averaged absolute values of *w* across healthy pianists, patients with FHD, and non-musicians, respectively. Shaded areas denote the standard error of the mean. **(B)** The absolute values of *w* for pieces #2-#9.

We calculated the weight coefficients *w* to confirm whether the motion components relevant to classifying playing tempi were prominent around the beginning of playing motion [as was done in a previous study (18)]. First, we reformatted *w* in each subject as *W* ∈ ℝ^15 × 200^ to discuss how the joint angles in each time frame contribute to classifying the playing tempo. Second, the current study calculated the absolute value of *W* because its amplitude rather than its sign represents the relevance of each joint angle to classifying the playing tempo. Third, we averaged the absolute values of *W* across all the joint angles to focus on temporal information. In the analysis of pieces #2-#9, the current study averaged *W* across all pieces. After these operations, the current study calculated the mean and standard error of the mean. Figure 3 validated our prediction: Motion components relevant to classifying the playing tempo into a natural tempo and the fastest one appeared especially around the beginning of the performance. Notably, the amplitude of *W* was subtle, indicating an evident difference between playing a musical piece at the fastest and natural tempi, especially in feedforward components, but this difference was embedded in a small portion of feedforward components. Along with the small amplitude of *W*, the norm of motion components that were relevant to classifying the playing tempo (the norm of $x_{k}^{rel}$ to be defined below) was 0.0467 in healthy pianists (averaged across all the subjects and pieces), and one irrelevant to classifying the playing tempo (the norm of $x_{k}^{irr}$ to be defined below) was 19.0. Because these tendencies were consistent among healthy pianists, patients with FHD, and non-musicians, the difference between playing a musical piece at the fastest tempo and a natural tempo was evident in a small portion of feedforward components.

To further discuss the kinematic features of playing at the fastest tempo, the current study extracted the motion components relevant to classifying data as being associated with either a natural tempo or the fastest tempo. Based on the functional form of $f\left(x_{k}\right)=\frac{1}{1+exp\left(-w_{0}-x_{k}w\right)}$, *w* affected *f*(*x*_*k*_) *via* *x*_*k*_*w*. The classification-relevant motion components $x_{k}^{rel}\in {\mathbb{R}}^{1\times 3000}$ can be written as,


(1) $$\begin{array}{r} x_{k}^{rel}=x_{k}\frac{ww^{T}}{|w{|}^{2}}\ldots . \end{array}$$

**Figure 4:**
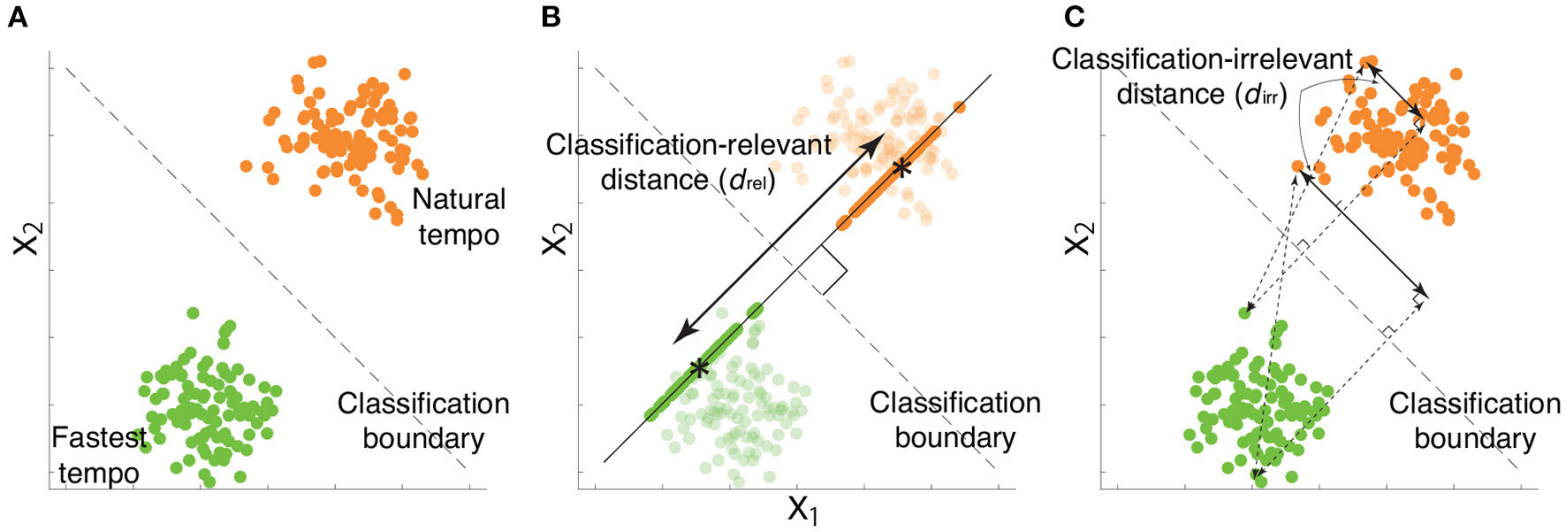
Examples of classification-relevant distance *d*_*rel*_ and classification-irrelevant distance *d*_*irr*_ based on simulated data. **(A)** Two-dimensional simulated data when considering a desired classification boundary (the black dotted line). Green and orange dots indicate simulated data from a single trial associated with the fastest and natural tempi, respectively. **(B)** Classification-relevant distance in the simulated data. The classification-relevant distance is based on the distance along the orthogonal direction to the classification boundary. Green and orange dots on the line orthogonal to the decision boundary indicate classification-relevant components. Asterisks within green and orange dots denote the averages of classification-relevant components associated with fastest and natural tempo, respectively. For example, if the distance between two groups of data along the direction is zero, classifying the data into fastest and natural tempi is impossible. **(C)** Classification-irrelevant distance in the simulated data. In the case of two-dimensional data, classification-irrelevant distance is based on the distance along the direction parallel to the classification boundary. If we observe two groups of data whose means are on the classification boundary but have different values, it is impossible to classify the data as reflecting either the fastest and natural tempi. Although the classification-relevant distance is zero in this case, the task-irrelevant distance along the classification boundary is not zero.

$x_{k}^{rel}$ is a portion of *x*_*k*_ such that $x_{k}w=x_{k}^{rel}w$ (i.e., $f\left(x_{k}\right)=f\left(x_{k}^{rel}\right)$) while avoiding the self-evident answer (i.e., $x_{k}^{rel}\neq x_{k}$) (22). It is possible to confirm that $x_{k}^{rel}$ has classification relevance by multiplying $x_{k}^{rel}$ by *w* from the right-hand side - $x_{k}^{rel}w=x_{k}\frac{ww^{T}}{|w{|}^{2}}w=x_{k}w$, indicating that $x_{k}^{rel}$ has information relevant to classifying the two tempi. In Figure 4A, the simulated motion data and decision boundary are shown as dots and a dotted line, respectively. Figure 4B denotes $x_{k}^{rel}$ as the motion data projected onto *w*, an orthogonal line to the decision boundary. Notably, $x_{k}^{rel}$ in Figure 4B possessed all information relevant to classifying the two categories.

Equation 1 indicates the orthogonal projection of *x*_*k*_ onto *w*, indicating that $x_{k}^{rel}$ has one dimension along the direction *w* (22). Due to the property of the orthogonal projection, $x_{k}^{rel}$ reflects the information of *w* to classifying playing tempi as either a natural tempo or the fastest tempo (Figure 4). $x_{k}^{rel}$ can thus be seen as a portion of feedforward components that are relevant to classifying playing tempi that are apparent around the beginning of playing motion. Hereafter, we refer to $x_{k}^{rel}$ as classification-relevant feedforward components.

Using $x_{k}^{rel}$, it is possible to define classification-irrelevant motion components as,


(2) $$\begin{array}{r} x_{k}^{irr}=x_{k}-x_{k}^{rel}\ldots . \end{array}$$

We can also confirm the classification irrelevance of $x_{k}^{irr}$ while multiplying $x_{k}^{irr}$ by *w* from the right-hand side - $x_{k}^{irr}w=x_{k}w-x_{k}^{rel}w=0$, i.e., $x_{k}^{irr}$ does not affect playing tempo classification. Because *w* takes small values (Figure 2), $x_{k}^{rel}$ explains a portion of feedforward components. In other words, $x_{k}^{irr}$ includes both feedforward and feedback components that are irrelevant to classifying playing tempi into a natural tempo and the fastest tempo. Hereafter, we refer to $x_{k}^{irr}$ as classification-irrelevant components.

By utilizing the classification-relevant feedforward components and classification-irrelevant components, we constructed a measure of the kinematic difference between playing at the natural and fastest tempi. Let us define $x_{n,k}^{rel}=x_{k}^{rel}$ and $x_{n,k}^{irr}=x_{k}^{irr}$ when *f*(*x*_*k*_) < 0.5 (i.e., the motion data at the *k*th were classified as the motions at the natural tempo), $x_{m,k}^{rel}=x_{k}^{rel}$ and $x_{m,k}^{irr}=x_{k}^{irr}$when *f*(*X*_*k*_) ≥ 0.5 (i.e., the motion data at the *k*th were classified as the motions at the fastest tempo), ${\tilde{T}}_{n}$ equaled the number of trials estimated to be associated with the natural tempo, and ${\tilde{T}}_{m}$ equaled the number of trials estimated to be associated with the fastest tempo (${\tilde{T}}_{n}+{\tilde{T}}_{m}=T_{n}+T_{m}$). Of note, ${\tilde{T}}_{n}\neq T_{n}$ and ${\tilde{T}}_{m}\neq T_{m}$ in most of the cases with misclassification(s). ${\tilde{T}}_{n}=T_{n}$ and ${\tilde{T}}_{m}=T_{m}$ only when all the motions were classified into the correct playing tempo. We then calculated the mean of the classification-relevant components associated with natural and fastest tempo as $\mu_{n}=\frac{1}{{\tilde{T}}_{n}}\overset{{\tilde{T}}_{n}}{\underset{k=1}{\sum }}x_{n,k}^{rel}$ and $\mu_{m}=\frac{1}{{\tilde{T}}_{m}}\overset{{\tilde{T}}_{m}}{\underset{k=1}{\sum }}x_{m,k}^{rel}$, respectively. We also calculated the variance averaged across the two conditions as $\sigma^{2}=\frac{1}{2}\left(\frac{1}{{\tilde{T}}_{n}}\overset{{\tilde{T}}_{n}}{\underset{k=1}{\sum }}\left(x_{n,k}^{rel}-\mu_{n}\right){\left(x_{n,k}^{rel}-\mu_{n}\right)}^{2}+\frac{1}{{\tilde{T}}_{m}}\overset{{\tilde{T}}_{m}}{\underset{k=1}{\sum }}\left(x_{m,k}^{rel}-\mu_{m}\right){\left(x_{m,k}^{rel}-\mu_{m}\right)}^{T}\right)$. Finally, the current study calculated *d*′ as a measure of the skill needed to play at the fastest tempo while focusing on classification-relevant feedforward motion components:


(3) $$\begin{array}{r} d_{rel}=d^{{}^{\prime }}=\sqrt{\frac{\left(\mu_{n}-\mu_{m}\right){\left(\mu_{n}-\mu_{m}\right)}^{T}}{\sigma^{2}}}\ldots . \end{array}$$

*d*′ is a measure based on the squared distance between *μ*_*n*_ and *μ*_*m*_ while considering the associated uncertainty (Figure 4B). A larger *d*_*rel*_ indicated a larger difference of classification-relevant feedforward components between the natural and fastest tempi, which probably indicated a faster motion at the fastest tempo compared to that at the natural tempo. Because *x*^*rel*^ was one dimensional (due to the property of orthogonal projection of *x* onto *w*), *d*_*rel*_ was a good measure of kinematic difference between two categories.

The current study also calculated a measure to quantify classification-irrelevant components. In contrast to *x*^*rel*^, whose dimension is one, *x*^*irr*^ has multiple dimensions. Because some eigenvalues of *x*^*irr*^ are close to 0 due to the low dimensionality inherent in motion data (22, 23, 27), the Mahalanobis distance or *d*′ is not an option for quantifying classification-irrelevant components. To quantify the difference in classification-irrelevant components between the two types of tempi, we calculated the following distance:


(4) $$\begin{array}{r} \begin{array}{c} d_{irr}=\sqrt{\frac{1}{{\tilde{T}}_{n}{\tilde{T}}_{m}}\sum_{k=1}^{{\tilde{T}}_{n}}\sum_{l=1}^{{\tilde{T}}_{m}}(x_{n,k}^{irr}-x_{m,l}^{irr}){\left(x_{n,k}^{irr}-x_{m,l}^{irr}\right)}^{T}}\ldots . \end{array} \end{array}$$

Equation 4 describes the group average method to calculate the distance between two clusters, utilized in hierarchical clustering methods. *d*_*irr*_ was thus an effective measure of the distance between the classification-irrelevant components at the natural and fastest tempi. Figure 4C demonstrates an example of *d*_*irr*_ in simulated motion data. *d*_*irr*_ is the distance between all pairs of classification-irrelevant components that belonged to different categories. A larger *d*_*irr*_ thus indicates a larger difference of classification-irrelevant components between the natural and fastest tempi. In the case of the two-dimensional data shown in Figure 4C, *d*_*irr*_ represents the distance along the decision boundary (i.e., black dotted lines in Figure 4). If there were the same tendency between *d*_*rel*_ and *d*_*irr*_ in the comparison among healthy pianists, patients with FHD, and non-musicians, skill learning would modulate not only classification-relevant feedforward components but also classification-irrelevant components. If there were different tendencies between *d*_*rel*_ and *d*_*irr*_ in the comparison among healthy pianists, patients with FHD, and non-musicians, skill learning would primarily affect classification-relevant feedforward components but would slightly affect classification-irrelevant components.

In sum, we calculated two measures to quantitatively assess the influence of skill learning on playing at two different tempi: one to quantify classification-relevant feedforward components based on Equation 3 and the other to quantify classification-irrelevant components based on Equation 4. For the joint angle analysis, we excluded 2 healthy pianists, 1 FHD patient, and 1 non-musician because multiple joint angles had not been measured in more than 10 trials. In total, we analyzed joint angle data from 11 healthy pianists, 23 patients with FHD, and 27 non-musicians (see STAR Methods for details).

**Figure 5:**
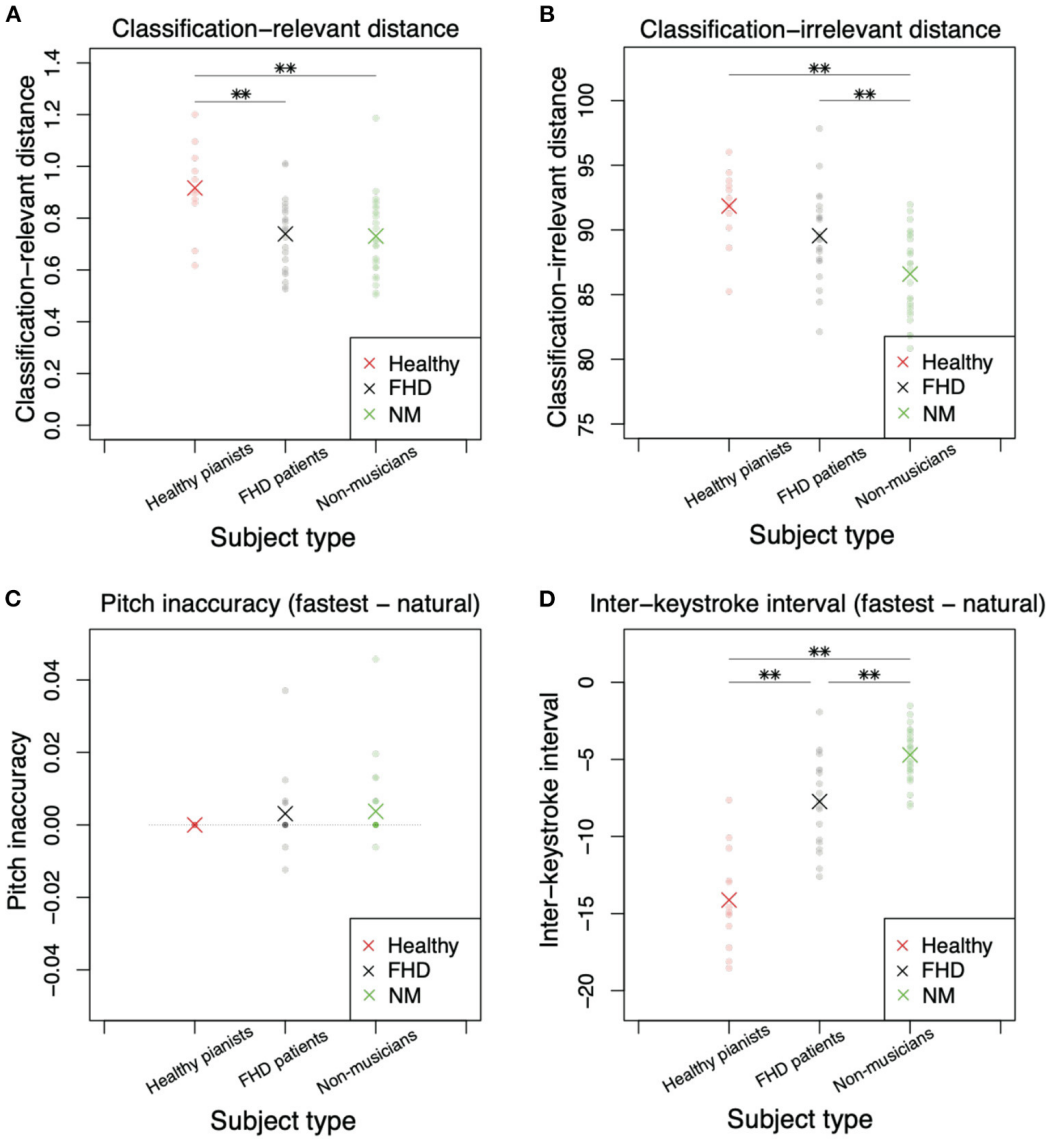
Comparisons of classification-relevant distance, classification-irrelevant distance, pitch inaccuracy, and interkeystroke interval among healthy pianists, patients with FHD, and non-musicians based on the simplest piece (piece #1). **(A)** Classification-relevant distance among the three groups. Red, black, and green dots denote the distances in each subject. Asterisks indicate mean classification-relevant distances in each group. Double asterisks indicate significant differences based on *p* < 0.01. **(B)** Classification-irrelevant distance. **(C)** Pitch inaccuracy in playing at the natural tempo subtracted from the inaccuracy in playing at the fastest tempo. **(D)** Interkeystroke interval in playing at the natural tempo subtracted from the inaccuracy in playing at the fastest tempo.

Figure 5 shows the results when subjects played piece #1 at the fastest and natural tempi. Figure 5A denotes *d*_*rel*_ (Equation 3), a measure of the difference in classification-relevant feedforward components between the natural and fastest tempi. Because playing a piece of music at a natural tempo can be regarded as a baseline skill in each subject, a larger *d*_*rel*_ indicates more sophisticated skill in playing music at the fastest tempo (i.e., a larger *d*_*rel*_ is likely associated with faster performance at the fastest tempo). Combined with the results shown in Figure 3, a larger *d*_*rel*_ denotes higher skill, especially around the beginning of playing motions. There was a significant main group effect on *d*_*rel*_ [F (2, 57) = 6.56, *p* = 0.00272]. We also found a significant difference in *d*_*rel*_ in healthy pianists compared to patients with FHD [p = 0.0065 (corrected)] and non-musicians [p = 0.0034 (corrected)]. However, there was no significant difference between patients with FHD and non-musicians [p = 1.00 (corrected)].

If both classification-relevant and classification-irrelevant motion components displayed the same tendency, there would be no difference in the trend between *d*_*rel*_ and *d*_*irr*_: A larger *d*_*irr*_ would be noted in healthy pianists compared to that observed in participants in the other groups. In contrast to this speculation, there was no significant difference in *d*_*irr*_ between healthy pianists and patients with FHD (Figure 5B, *p* = 0.180 [corrected]). Additionally, there were significant differences between patients with FHD and nonmusicians [p = 0.00747 (corrected)] and between healthy pianists and non-musicians [p = 0.00011 (corrected)]. In addition, there was a main group effect [F (2, 57) = 11.3, p = 7.32 × 10^−5^].

In summary, the joint angle analysis revealed different tendencies between classification-relevant feedforward components and classification-irrelevant components. The differences in these components between healthy pianists and non-musicians were evident (Figures 5A,B). Of note, skill learning enables healthy pianists to play at the fastest tempo in a more sophisticated manner. In the comparison of healthy pianists to patients with FHD, classification-relevant feedforward components were altered due to maladaptation (Figure 5A). Patients with FHD showed the same tendency of classification-relevant feedforward components as non-musicians (Figure 5A). In contrast, patients with FHD showed classification-irrelevant components comparable to those of healthy pianists (Figure 5B). Notably, small amplitudes of **w** resulted in small classification-relevant distances. We thus concluded that maladaptation affects classification-relevant feedforward components.

A potential problem inherent in the analysis of joint angle data is the difficulty in discussing accuracy and playing speed while comparing two different tempi. Normalization of movement time should be required to compare the kinematics between the two tempi, resulting in the elimination of the detailed information about movement time or interkeystroke interval. In addition to movement time, joint angle data do not explicitly include accuracy information in playing musical pieces. Although motor skill is defined by (at least) speed and accuracy, joint angle analysis is insufficient to describe motor skill. To quantitatively validate a difference in skill levels of the participants across the groups, the current study utilized keypress data representing motions of the depression and release of the keys. Through the validation of skill level *via* keypress data analysis, the results of joint angle data analysis can be associated with motor skill.

We focused on two variables: the probability of an incorrect touch (pitch inaccuracy) and interkeystroke interval (movement time) at natural and the fastest tempi. In the case of piece #1, the probability of incorrect touch was zero without any variability in healthy pianists. We thus calculated a simple difference in the probability of incorrect touch between natural and fastest tempi as a measure of motor skill to play at the fastest tempo. To utilize the same measure in the keypress data analysis, we calculated a simple difference in the averaged interkeystroke intervals between the natural and fastest tempi. After calculating the difference in each variable in each subject between the two tempi, we compared the data among the three groups. For the keypress data analysis, we excluded subjects whose data had also been excluded from the analysis of finger joint angles.

Keypress data analysis enabled us to clarify the skill level in each group based on the perspectives of the speed-accuracy tradeoff. In pitch inaccuracy, there was no significant main group effect [Figure 5C, F (2, 49) = 0.504, *p* = 0.607]. We did not find any significant difference among the groups (*p* > 0.96, *t* test with Bonferroni's correction).

Under the same level of accuracy, there was a significant main group effect for the interkeystroke interval [Figure 5D, F (2, 57) = 63.2, *p* = 3.49 × 10^−15^]. We found a significant difference in the interkeystroke interval among the groups (Figure 5D, *p* = 6.1 × 10^−9^ [corrected] between healthy pianists and patients with FHD, p = 1.3 × 10^−15^ [corrected] between healthy pianists and non-musicians, and *p* = 0.00044 [corrected] between patients with FHD and non-musicians). Figure 5D denotes the distance of the interkeystroke interval from the natural tempo to the fastest tempo. A negative value indicated a faster performance at the fastest tempo than at the natural tempo (i.e., shorter interkeystroke intervals at the fastest tempo than at the natural tempo). Healthy pianists showed the largest difference in the interkeystroke intervals between the two tempi, indicating that they performed the fastest piano-playing motions among the three groups. Non-musicians showed the smallest difference in the interkeystroke intervals between the two tempi, indicating fewer rapid motions even at the fastest tempo. Notably, patients with FHD showed a larger difference in the interkeystroke intervals between the two tempi than non-musicians; however, the tempowise difference was smaller in patients with FHD than in healthy pianists. Patients with FHD thus played the piano faster than non-musicians but slower than healthy pianists at the fastest tempo. From the perspective of the speed-accuracy tradeoff, patients with FHD showed more sophisticated skill than non-musicians; nevertheless, patients with FHD were not comparable to healthy practitioners.

The results mentioned above were based on one of the simplest pieces of music. It remained unclear whether the results were invariant for more difficult pieces of music. We thus further examined the same measures in more difficult pieces of music (the details of the pieces are provided in the Supplementary material). Because these pieces were too difficult for non-musicians to play at the fastest tempo, the following analyses were performed for healthy pianists and patients with FHD.

**Figure 6:**
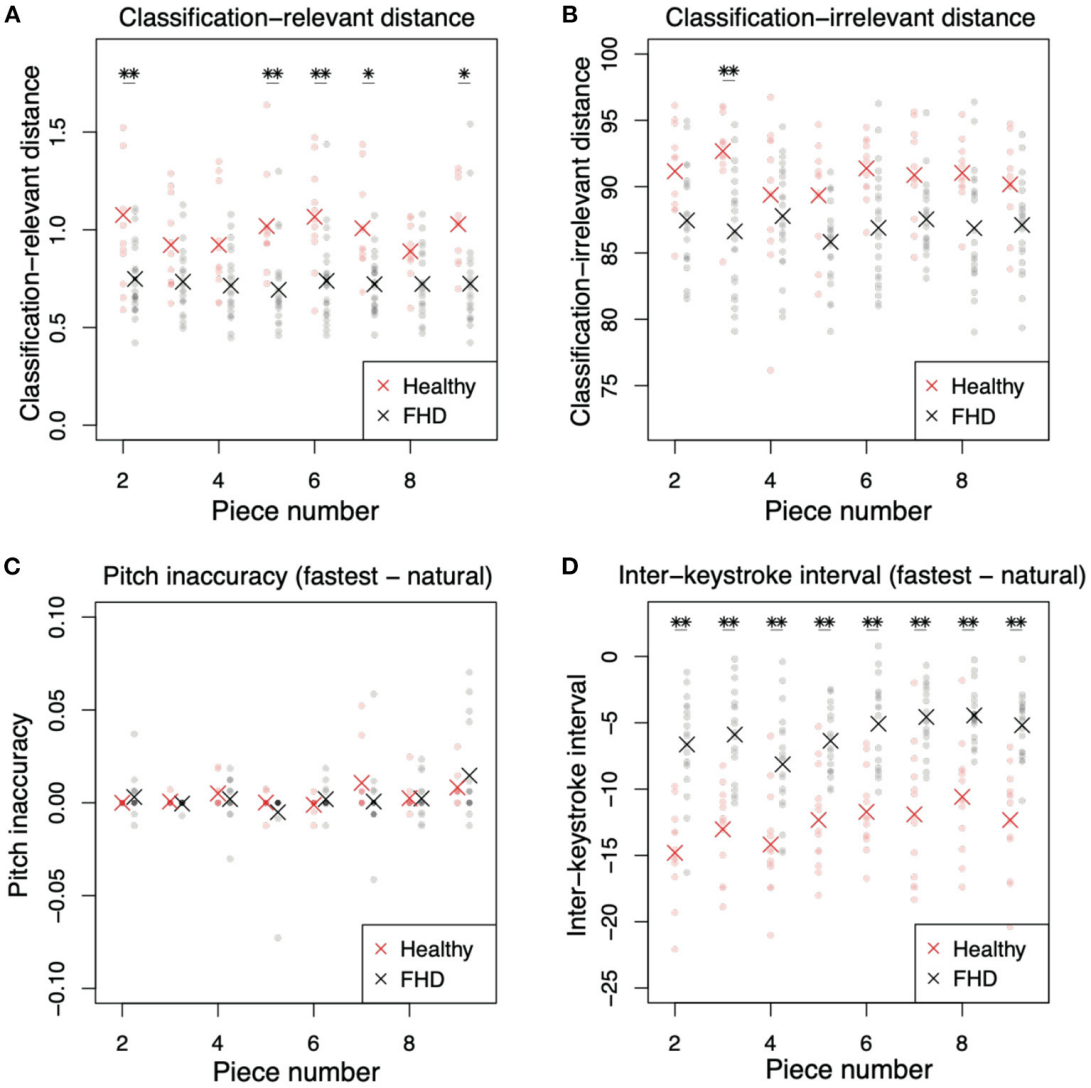
Comparisons of classification-relevant distance, classification-irrelevant distance, pitch inaccuracy, and interkeystroke interval between healthy pianists and patients with FHD for pieces #2-#9. **(A)** Classification-relevant distance between participants in the two groups. Red and black dots denote the distances in each subject. Asterisks indicate mean classification-relevant distances in each group. Single and double asterisks indicate significant differences based on *p* < 0.05 and *p* < 0.01, respectively. **(B)** Classification-irrelevant distance. **(C)** Pitch inaccuracy in playing at the natural tempo subtracted from the inaccuracy in playing at the fastest tempo. **(D)** Interkeystroke interval in playing at the natural tempo subtracted from the inaccuracy in playing at the fastest tempo.

The kinematics results in more difficult pieces had varying tendencies compared to those of the simplest piece (Figure 6). In the classification-relevant difference *d*_*rel*_ (Figure 6A), there was a significant main group effect [F (1, 32) = 33.6*, p* = 1.98 × 10^−6^], no significant main effect of piece number [F (7, 224) = 0.764, *p* = 0.618], and no significant interaction between group and piece number [F (7, 224) = 0.961, *p* = 0.461]. The classification-relevant difference in healthy pianists was larger than that in patients with FHD in more than half the pieces (Figure 6A, *p* < 0.0337 [corrected] in pieces 2, 5, 6, 7, and 9, and p > 0.388 [corrected] in other pieces). These results were similar to the result in the simplest piece of music.

In classification-irrelevant distance *d*_*irr*_ (Figure 6B), there was a significant main group effect [F (1, 32) = 11.9, p = 1.62 × 10^−3^], no significant main effect of piece number [F (7, 224) = 1.68, *p* = 0.114], and no significant interaction between group and piece number [F (7, 224) = 2.00, *p* = 0.0558]. In piecewise comparisons between the two groups in each piece, there was a significant difference between healthy pianists and patients with FHD in piece 3 (Figure 6B, *p* = 0.00320 [corrected]), but there was no significant difference in the other pieces (*p* > 0.104 [corrected]).

The results of keypress data analysis for more difficult pieces (pieces #2-#9) were similar to those of the simplest piece (pieces #1). In pitch inaccuracy (Figure 6C), there was no significant main group effect [F (1, 24) = 0.161, *p* = 0.692], a significant main effect of piece number [F (7, 168) = 3.35, *p* = 2.26 × 10^−3^], and no significant interaction [F (7, 168) = 1.11, *p* = 0.367]. The main effect of piece number indicated that the difference in difficulty in each piece affected pitch inaccuracy. In piecewise comparisons, there was no difference between healthy pianists and patients with FHD (*p* > 0.853 [corrected]). These results were the same as those for the simplest piece—no difference in pitch accuracy between the groups.

In the analysis of the interkeystroke interval (Figure 6D), there were significant main effects of group [F (1, 30) = 52.3, *p* = 4.73 × 10^−8^] and piece number [F (7, 210) = 9.09, *p* = 8.43 × 10^−10^], but there was no significant interaction between these factors [F (7, 210) = 0.849, *p* = 0.548]. In piecewise comparisons, there were significant differences between healthy pianists and patients with FHD in all pieces (*p* < 5.99 × 10^−4^ [corrected]). The main effect of piece number indicated the influence of difficulty of musical pieces (i.e., task difficulty) on the interkeystroke intervals. We found the same results as those observed for the simplest piece—healthy pianists showed faster interkeystroke intervals than patients with FHD.

## Discussion

We compared the finger joint angles and keypress data among healthy pianists, patients with FHD, and non-musicians when they played one of the simplest pieces of music at the natural or fastest tempo. The current study detected the joint angle features relevant to classifying the fastest and natural tempi (Equations 3, 4). The classification-relevant motion components were evident around the beginning of playing motions (Figure 3). While considering the small amplitudes of the classification-relevant motion components, a small portion of feedforward components played roles in classifying playing tempo into natural and fastest ones. In other words, a large portion of feedforward and feedback motion components were irrelevant to classifying playing tempo. Healthy pianists showed differences from patients with FHD and non-musicians in the classification-relevant feedforward motion components (Figure 5A). The lack of significant difference in classification-relevant feedforward components between patients with FHD and non-musicians indicated that classification-relevant feedforward motion components were impaired specifically in patients with FHD, possibly due to maladaptation of the sensorimotor system. In contrast, there was no significant difference in classification-irrelevant motion components between healthy pianists and patients with FHD (Figure 5B). When playing at the fastest tempo, non-musicians demonstrated different tendencies in classification-irrelevant motion components compared to both healthy pianists and patients with FHD. Additionally, the current study examined how the technical difficulty inherent in playing piano pieces affects these results (Figures 6A,B). Although the difficulty affected the results to some degree, the trend in our results was consistent independent of the difficulty.

**Figure 7:**
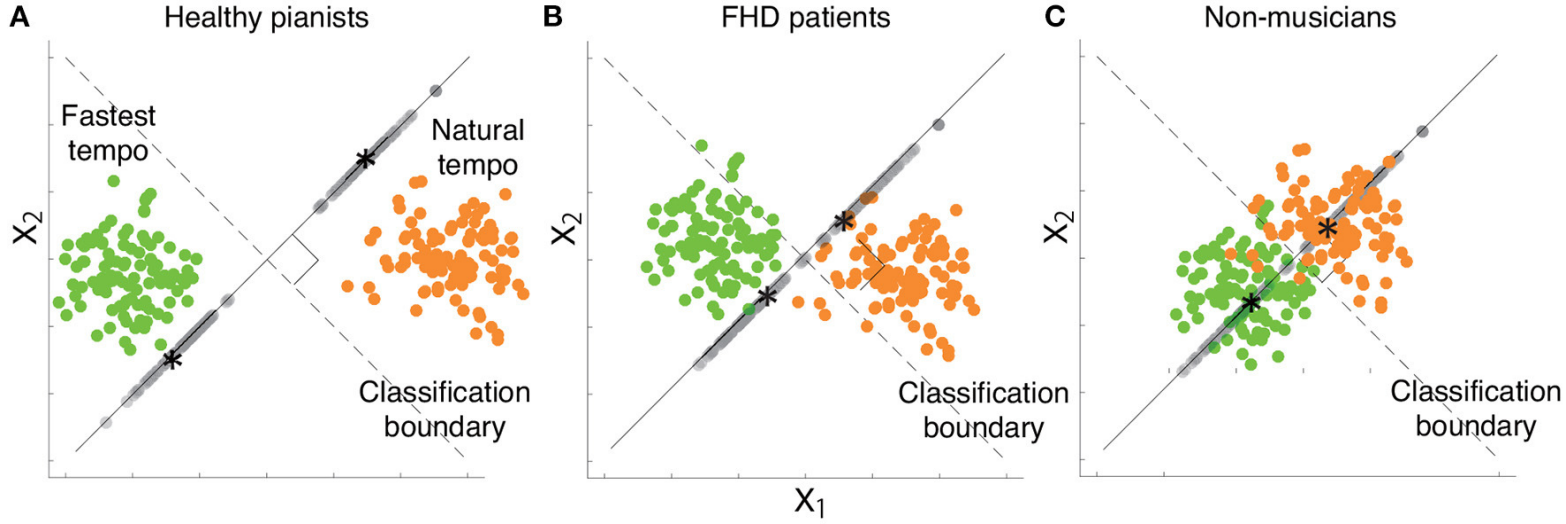
Schema of our results based on two-dimensional simulated data while considering a desired classification boundary (the black dotted line). Green and orange dots indicate simulated data from a single trial associated with the fastest and natural tempi, respectively. Gray dots denote each data point projected onto a line orthogonal to the classification boundary. Black asterisks indicate the mean of the projected data associated with the fastest or natural tempo. **(A)** Simulated data that indicate kinematic features in healthy pianists. **(B)** Simulated data that indicate kinematic features in patients with FHD. **(C)** Simulated data that indicate kinematic features in non-musicians.

Figure 7 summarizes the current results based on simulated motion data. Playing the piano at various tempi from slow (e.g., largo in musical notes) to fast tempo (e.g., prestissimo) is an essential element of expression for pianists. Therefore, pianists are well-trained to play the piano at the natural and fastest tempi. The effect of extensive training is observable *via* the comparison between healthy pianists (Figure 7A) and non-musicians (Figure 7C). Our results suggest that extensive training enables healthy pianists to achieve clear separation of the two tempi in joint angle data, i.e., they demonstrate sophisticated classification-relevant feedforward components while considering the results shown in Figure 3. Despite maladaptation, patients with FHD are also well trained to play at different tempi. We confirmed the effects of maladaptation through a comparison between healthy pianists and patients with FHD (Figure 7B). Patients with FHD modulated their finger movements depending on tempo at the same level as healthy pianists, which can be confirmed based on *d*_*irr*_ (Figures 5B, 6B). However, the modulation did not correspond with the dimension relevant to changing the tempo (Figure 7B). The impaired tempo-dependent modulations of joint angle motions were associated with impaired classification-relevant feedforward motion components (Figure 3). In sum, our results suggest that extensive training enables pianists to modulate their finger movements to distinguish different tempi but that maladaptation locks the tempo-dependent modulation away from the classification-relevant dimension.

From the perspectives of keypress data analysis, patients with FHD showed a prolonged interkeystroke interval compared with that of healthy pianists (Figures 5D, 6D). Non-musicians demonstrated a larger interkeystroke interval than patients with FHD. Moreover, for pitch accuracy, there was no significant difference among healthy pianists, patients with FHD, and non-musicians (Figures 5C, 6C). Due to the same level of accuracy and slowed movement tempo, patients with FHD had worse motor skill levels than healthy pianists based on the speed-accuracy tradeoff. Despite the impaired skill level, patients with FHD still demonstrated better motor skill levels than non-musicians.

Although our results support the impairment of some feedforward control in patients with FHD, it remains unclear why such impairment occurs in patients with FHD. In patients with embouchure dystonia, one form of task-specific dystonia, cerebellar malfunctions were related to dystonic symptoms (38). In pianists with FHD, cerebellar activities and the functional connections between the cerebellum and premotor/somatosensory cortex differed from those of healthy pianists (39). In addition to these studies based on functional magnetic resonance imaging (fMRI), several studies have proposed the relation of the cerebellum to feedforward control from behavioral and computational perspectives (10–14). We thus speculate that cerebellar malfunctions are associated with the impairment of feedforward control in patients with FHD. Notably, our results indicated the impairment of classification-relevant feedforward control, i.e., a fraction of feedforward control, rather than whole feedforward control. The difference in cerebellar activities between patients with FHD and healthy pianists thus could be associated with the impairment of classification-relevant feedforward control.

In sum, we clarified some aspects of the effects of skill learning and maladaptation on motor skill. Through the comparison between healthy pianists and non-musicians, skill learning was shown to facilitate appropriate finger motions for playing musical pieces at the fastest tempo [i.e., a larger classification-relevant difference (Figure 5A) and with a faster interkeystroke interval (Figure 5D) in healthy pianists than in non-musicians]. Although skill learning also modulated finger movements irrelevant to classifying playing tempo (Figure 5B), the effects of skill learning on movement accuracy were not evident in the playing of one of the simplest musical pieces (Figure 5C). As illustrated by the comparison between healthy pianists and patients with FHD, this disorder impaired the dexterous finger motions needed to play musical pieces at the fastest tempo [i.e., a smaller classification-relevant difference (Figures 5A, 6A) and generates a larger interkeystroke interval (Figures 5D, 6D) in patients with FHD than in healthy pianists]. Interestingly, FHD did not affect a large portion of motions irrelevant to classifying playing tempi (Figures 5B, 6B) despite impaired motion features relevant to classifying playing tempi. Because classification-relevant feedforward motion components consisted of a small portion of movements, it would be difficult to diagnose patients with FHD based solely on finger kinematics. In contrast, there was a clear difference in movement time (i.e., interkeystroke interval) between healthy pianists and patients with FHD. Our results thus indicated that movement time is an efficient and reliable measure to aid in diagnosing FHD.

## STAR methods

### Participants

Twenty-four pianists with unilateral musician's dystonia (MD) (age 39.5 ± 10.6 years; 10 males, all right-handed pianists), 13 age-matched healthy pianists who had undergone formal musical education and training at conservatories (age 35.5 ±11.6 years; 2 males, all right-handed pianists), and 28 age-matched healthy individuals with no history of piano training (i.e., non-musicians, age 27.4 ± 8.3 years; 7 males) participated in the study. We studied MD as a model of FHD for the following two reasons. First, while musical performance requires more dexterous movement control and carries a higher risk for developing FHD than other tasks, such as handwriting (40), little is known about the pathological mechanism of MD. Second, methods have been established to quantitatively assess motor dexterity during piano playing (27). All pianists underwent a thorough neurological examination. The exclusion criteria were bilateral FHD; generalized dystonia; epilepsy; a history of any other neurological diseases; and a history of pharmacological intervention, including neuroleptic drugs and the injection of botulinum toxin, within at least the past 6 months. The participants had no histories of other neuropsychiatric disorders, neurosurgery, or metal or electronic implants. The symptoms entailed exaggerated finger flexion or thumb adduction in most cases and difficulty of finger extension due to an involuntary flexor cramp pulling the finger(s) down in some cases. None of the patients reported a family history of FHD. In accordance with the Declaration of Helsinki, the experimental procedures were explained to all participants. Informed consent was obtained from all participants prior to participation in the experiment, and the whole experimental protocol was approved by the Ethics Committee of Sophia University (18-F-0001).

### Data processing

We measured the joint angle data (see Table 1 for the measured joints) by using the CyberGlove III (CyberGlove Systems) at 120 Hz with angular resolution <0.5 degrees. We excluded joint angle data from 2 healthy pianists, 1 FHD patient, and 1 non-musician because there was no within-trial variation in some joint angles (due to measurement error). We also excluded keypress data from the same subjects to maintain analysis consistency.

First, each joint angle data point was normalized to have 10,000 time frames irrespective of playing tempo, which enabled us to analyze the data measured under different movement times. Second, the normalized joint angle data were low-pass filtered at 15 Hz using a 5th-order Butterworth filter. Finally, to shorten the computational time in logistic regression, we further formatted each joint angle data to have 200 time frames.

### Statistical analysis

For the comparison among healthy pianists, patients with FHD, and non-musicians in piece #1, we performed ANOVA for each logarithmic classification error (Figure 2) or distance (Figures 5, 6) with the error or distance as a dependent variable and the group index as an independent variable. For the comparison between healthy pianists and patients with FHD based on eight pieces of music, we performed a mixed model ANOVA analysis with the error or distance as a dependent variable and the group index as an independent variable; the piece number was an independent variable measured repeatedly within subjects. All of the *post-hoc* pairwise and multiple comparisons were based on Tukey's HSD to correct the effects of multiple comparisons.

## Data availability statement

The raw data supporting the conclusions of this article will be made available by the authors, without undue reservation.

## Ethics statement

The studies involving human participants were reviewed and approved by the Ethics Committee of Sophia University (18-F-0001). The patients/participants provided their written informed consent to participate in this study.

## Author contributions

KT, SM, and SF contributed to the conception and design of the study and wrote sections of the manuscript. SF organized the database. KT and SM performed the statistical analysis. KT wrote the first draft of the manuscript. All authors contributed to manuscript revision, read, and approved the submitted version.

## Funding

This study was supported by a Grant-in-Aid for Scientific Research (B) (20H04089) to KT and by JST CREST (JPMJCR17A3), JSPS Grant-in-Aid for Transformative Research Areas B (20H05713), and Fostering Joint International Research (B) (19KK0252) to SF.

## Conflict of interest

Author SF was employed by Sony Computer Science Laboratories Inc. The remaining authors declare that the research was conducted in the absence of any commercial or financial relationships that could be construed as a potential conflict of interest.

## Publisher's note

All claims expressed in this article are solely those of the authors and do not necessarily represent those of their affiliated organizations, or those of the publisher, the editors and the reviewers. Any product that may be evaluated in this article, or claim that may be made by its manufacturer, is not guaranteed or endorsed by the publisher.
