# Supplementary material for: Impaired feedforward control of movements in pianists with focal dystonia
Source: Front Neurol. 2022 Aug 12;13:983448. doi: 10.3389/fneur.2022.983448 (PMC9413149; doi:10.3389/fneur.2022.983448)
Supplement: Supplementary file 1 [file Data_Sheet_1.docx]

**Supplementary Information**

Title:

Impaired feedforward control of movements in pianists with focal dystonia

Authors:

Ken Takiyama, Syuta Mugikura and Shin-ichi Furuya

Fig. S1: Pieces #2-#9. Upper-left numbers denote piece numbers. The numbers below musical notes indicate the fingers to use to play each note: 1 indicates the thumb, 2 indicates the index finger, 3 indicates the middle finger, 4 indicates the ring finger, and 5 indicates the little finger.
